# Multidrug resistance and biofilm formation among bacterial isolates from chronic wounds in animals: evaluation of anti-biofilm activity

**DOI:** 10.3389/fvets.2026.1916814

**Published:** 2026-08-31

**Authors:** Safia Arbab, Hanif Ullah, Suleman, Mazen Almehmadi, Mamdouh Allahyani, Abdulelah Aljuaid, Khalid Jambi, Mustafa H. Halawi, Chao Feng

**Affiliations:** 1 Medicine and Engineering Interdisciplinary Research Laboratory of Nursing & Materials/Nursing Key Laboratory of Sichuan Province, West China Hospital, Sichuan University/West China School of Nursing, Sichuan University, Chengdu, Sichuan, China; 2 Lanzhou Institute of Husbandry and Pharmaceutical Sciences, Chinese Academy of Agricultural Sciences, Lanzhou, China; 3 Department of Zoology, Government Post Graduate College, Swabi, Pakistan; 4 Higher Education Department, Civil Secretariat Peshawar, Peshawar, Pakistan; 5 Department of Clinical Laboratory Sciences, College of Applied Medical Sciences, Taif University, Taif, Saudi Arabia; 6 Medical Laboratory Technology Department, College of Nursing and Health Sciences, Jazan University, Jazan, Saudi Arabia; 7 West China Hospital, Sichuan University, Chengdu, China

**Keywords:** antimicrobial susceptibility, biofilm, chronic wound infections, MIC, multidrug resistance, veterinary microbiology

## Abstract

Chronic wound infections in animals represent a growing clinical challenge due to the emergence of multidrug-resistant (MDR) bacteria and their ability to form biofilms, which significantly reduce treatment efficacy and promote persistent infection. This study aimed to characterize the bacterial profile, antimicrobial resistance patterns, biofilm-forming capacity, and anti-biofilm activity of agents against bacterial isolates recovered from chronic wound specimens. A total of 96 wound samples were analyzed, of which 84 (87.5%) yielded positive bacterial growth. The predominant bacterial isolates were *Staphylococcus aureus* (33.3%), followed by *Pseudomonas aeruginosa* (27.4%), *Escherichia coli* (19.0%), and *Klebsiella* spp. (11.9%). Antimicrobial susceptibility testing revealed high resistance to β-lactam antibiotics, with ampicillin showing the highest resistance rate (78.6%), whereas imipenem exhibited the lowest resistance (14.3%). Overall, 63.5% of isolates were classified as multidrug-resistant. Biofilm analysis demonstrated that 38.1% of isolates were strong biofilm producers, with strong biofilm-forming strains exhibiting higher minimum inhibitory concentration (MIC) values compared with weak and non-biofilm producers. Evaluation of anti-biofilm agents showed that silver nanoparticles, chitosan nanoparticles, and DNase enzyme reduced biofilm biomass by 72%, 65%, and 58%, respectively. These findings highlight the critical role of biofilm-mediated resistance in chronic wound infections and emphasize the need for integrated antimicrobial and anti-biofilm strategies for effective management of MDR bacterial infections.

## 1 Introduction

Chronic wound infections are a major issue in veterinary medicine and occur in many types of animals (livestock and pets). Trauma, surgical problems, parasitism, and systemic diseases are all potential causes of these wounds (1). Chronic wounds would not heal properly; they would remain in the inflammatory stage, while an acute wound would go through the normal stages of healing. Persistent microbial colonization and the production of biofilms are one of the major reasons for this impaired wound healing (2, 3).

Biofilms are organized groups of microorganisms that are attached to living surfaces and surrounded by an extracellular polymeric substance (EPS) matrix. This matrix shields bacteria in the biofilm from environmental stressors, the host immune system, and antimicrobials, and enables them to survive in environments that would be lethal when not inside the matrix (4). Therefore, biofilm bacteria are much harder to treat than planktonic bacteria, with infections being more difficult to eradicate (5, 6).

Various bacterial species are commonly isolated in cases of long-term wound infection in veterinary clinical practice. They typically harbor the presence of *Staphylococcus aureus, Pseudomonas aeruginosa, Escherichia coli*, and *Klebsiella* spp, and each of these has virulence factors that help them attach, survive, and evade the immune system (2, 7, 8). Besides the ability to form biofilms, many of these organisms have developed multidrug resistance (MDR), making management of these organisms more difficult, as well as raising concerns regarding the efficacy of treatment and the possibility of zoonotic transmission of resistant strains.

The classical methodologies of antimicrobial susceptibility testing, including disk diffusion and determination of minimum inhibitory concentration (MIC), continue to be fundamental tools in clinical microbiology to guide therapy. These methods are, however, mainly used to measure planktonic populations of bacteria and may not be accurate in measuring the resistance profile of the biofilm-embedded bacteria (9, 10). This restriction highlights the importance of considering chronic wound pathogens in an integrated fashion, including the ability to form biofilms as well as antimicrobial resistance.

In recent years, research has increasingly focused on novel anti-biofilm strategies to enhance treatment outcomes in chronic wound care. They include products such as nanoparticles, enzymes that break down the matrix of the biofilm, and natural compounds that have the ability to inhibit the biofilm (11). These methods may prove to complement traditional antimicrobials and overcome the constant problem of biofilm-related infections.

Therefore, the present study was undertaken to investigate the prevalence, antimicrobial resistance pattern, and biofilm-forming property of bacterial isolates from chronic wound infections of animals. Furthermore, the present study evaluated the effectiveness of the selected anti-biofilm agents to combat these stubborn infections.

## 2 Materials and methods

### 2.1 Ethical approval

This study involved the collection and analysis of clinical wound specimens from patients with chronic, non-healing wounds. The study protocol was reviewed and approved by the Institutional Ethics Committee of the Lanzhou Institute of Husbandry and Pharmaceutical Sciences, Chinese Academy of Agricultural Sciences. Written informed consent was obtained from all participants before sample collection. All procedures were performed in accordance with relevant ethical guidelines and institutional regulations.

### 2.2 Study design and sample collection

This prospective laboratory-based study was conducted over a period of 6 months. A total of 96 clinical wound specimens were collected from patients with chronic, non-healing wounds showing clinical signs of infection, including exudation, inflammation, and delayed wound healing.

Before sample collection, wound surfaces were gently cleaned with sterile normal saline to minimize superficial contamination. Samples were collected aseptically from the deeper wound area using sterile swabs following the Levine sampling technique to preferentially obtain clinically relevant pathogenic microorganisms rather than superficial colonizing bacteria. The collected specimens were immediately transported to the microbiology laboratory under appropriate conditions for microbiological analysis and further processing.

### 2.3 Isolation and culture of bacterial isolates

Collected samples were cultured on Blood agar and MacConkey agar, aerobically at 37 °C for 18–24 h. Following incubation, separate colonies were picked and subcultured several times to get pure bacterial isolates. All standard microbiological precautions were taken during the isolation period.

### 2.4 Isolation and identification of bacterial isolates

Bacterial identification was carried out by the colony morphology method. Bacteriological identification was performed by colony morphology, Gram staining, and routine biochemical tests. This included catalase, coagulase, oxidase, indole production, citrate utilization, urease activity, and triple sugar iron (TSI) agar reactions.

*Staphylococcus aureus* was found to be catalase- and coagulase-positive Gram-positive cocci. *P. aeruginosa* was identified as Gram-negative rods, which were oxidase- and citrate-positive. *Escherichia coli* was indole + and citrate-, whereas *Klebsiella* spp. was urease+ and citrate +. The biochemical characteristics of the organisms are summarized in (Table 1).

**Table 1:** Biochemical characteristics of major bacterial isolates recovered from chronic wound infections.

Test	*Staphylococcus aureus*	*Pseudomonas aeruginosa*	*Escherichia coli*	*Klebsiella* spp.
Gram staining	Gram-positive cocci	Gram-negative rods	Gram-negative rods	Gram-negative rods
Catalase	+	+	+	+
Coagulase	+	–	–	–
Oxidase	–	+	–	–
Indole	–	–	+	–
Citrate utilization	–	+	–	+
Urease	–	–	–	+
TSI reaction	Yellow/yellow (A/A)	Red/red (K/K)	Yellow/yellow (A/A)	Yellow/yellow (A/A)

+, Positive reaction; –, Negative reaction; TSI, Triple Sugar Iron agar; A/A, Acid slant/Acid butt; K/K, Alkaline slant/Alkaline butt.

### 2.5 Antimicrobial susceptibility testing

#### 2.5.1 Kirby-Bauer disk diffusion method

Antimicrobial susceptibility testing was performed using the Kirby–Bauer disk diffusion method on Mueller–Hinton agar according to the guidelines of the Clinical and Laboratory Standards Institute (CLSI). Bacterial suspensions were adjusted to a 0.5 McFarland turbidity standard and evenly spread onto the agar surface.

The following antibiotics (with standard disc potencies) were tested: ampicillin, amoxicillin-clavulanic acid, ceftriaxone, ceftazidime (CAZ), ciprofloxacin, levofloxacin, gentamicin, amikacin, tetracycline, and imipenem. The plates were incubated aerobically at 37 °C for 18–24 h. Zones of inhibition were measured in millimeters and interpreted as susceptible, intermediate, or resistant according to CLSI breakpoint criteria (12).

### 2.6 Determination of minimum inhibitory concentration (MIC)

The minimum inhibitory concentrations (MICs) were determined by the broth microdilution method using sterile 96-well microtiter plates according to CLSI guidelines. Antibiotic dilutions were prepared by serial two-fold dilution in Mueller–Hinton broth. Standardized bacterial inoculates were added to each well, including growth control wells (without antibiotics) and sterility control wells (without bacteria). Plates were incubated at 37 °C for 18–24 h, and the MIC was defined as the lowest antibiotic concentration showing no visible growth. MIC values were determined using planktonic bacterial cultures and were interpreted as indicators of antimicrobial susceptibility of bacterial isolates rather than direct measurements of biofilm-associated resistance.

### 2.7 Biofilm formation assay

The ability of the isolates to form biofilms was determined using the microtiter plate assay. Overnight bacterial cultures were diluted in nutrient broth supplemented with glucose and inoculated into sterile 96-well microtiter plates. The plates were incubated under appropriate conditions to allow biofilm formation. After the incubation period, wells were gently washed with phosphate-buffered saline to remove non-adherent planktonic cells. The remaining attached biofilm biomass was stained with 0.1% crystal violet solution, followed by washing to remove excess stain. The bound crystal violet was solubilized using ethanol, and the optical density was measured at 570 nm. Based on the absorbance values, isolates were classified as non-, weak, moderate, or strong biofilm producers. Biofilm formation capacity was evaluated independently from MIC determination to assess the ability of bacterial isolates to produce adherent biofilm biomass under laboratory conditions.

### 2.8 Determination of multidrug resistance (MDR)

Multidrug resistance was defined as resistance to at least three antimicrobial classes according to standardized criteria. MDR classification was based on antimicrobial susceptibility results obtained from disk diffusion testing, supported by MIC data where applicable. Intermediate susceptibility was not considered resistant for MDR categorization.

### 2.9 Evaluation of anti-biofilm activity

Strong biofilm-producing isolates were selected for anti-biofilm evaluation. Pre-formed biofilms were established in sterile 96-well microtiter plates and treated with silver nanoparticles, chitosan nanoparticles, and DNase enzyme at predetermined concentrations. Untreated biofilms were maintained as negative controls, while wells without bacterial inoculation served as sterility controls. After treatment and incubation under standardized conditions, the remaining biofilm biomass was quantified using the crystal violet microtiter plate assay described previously. The percentage of biofilm reduction was calculated by comparing the optical density values of treated biofilms with those of untreated controls. All experiments were performed in triplicate, and results were expressed as mean ± standard deviation. Nanoparticle characterization parameters, including particle size, morphology, and surface properties, were recorded based on previously established characterization methods.

### 2.10 Statistical analysis

All experiments were performed in triplicate, and results were expressed as mean ± standard deviation (SD). Statistical analysis was performed using Microsoft Excel (Microsoft Corporation, USA). Group comparisons were evaluated using one-way analysis of variance (ANOVA) followed by Tukey's multiple comparison test. The Chi-square test was used to analyze associations between categorical variables, including biofilm formation categories and multidrug resistance status. A *p*-value < 0.05 was considered statistically significant.

## 3 Results

### 3.1 Bacterial isolation and prevalence

A total of 96 chronic wound specimens were analyzed, of which 84 (87.5%) yielded positive bacterial growth, indicating a high prevalence of bacterial infection among chronic wound patients. The percentage of Gram-negative bacteria was 54.8%, while Gram-positive bacteria were 45.2%.

**Figure 1:**
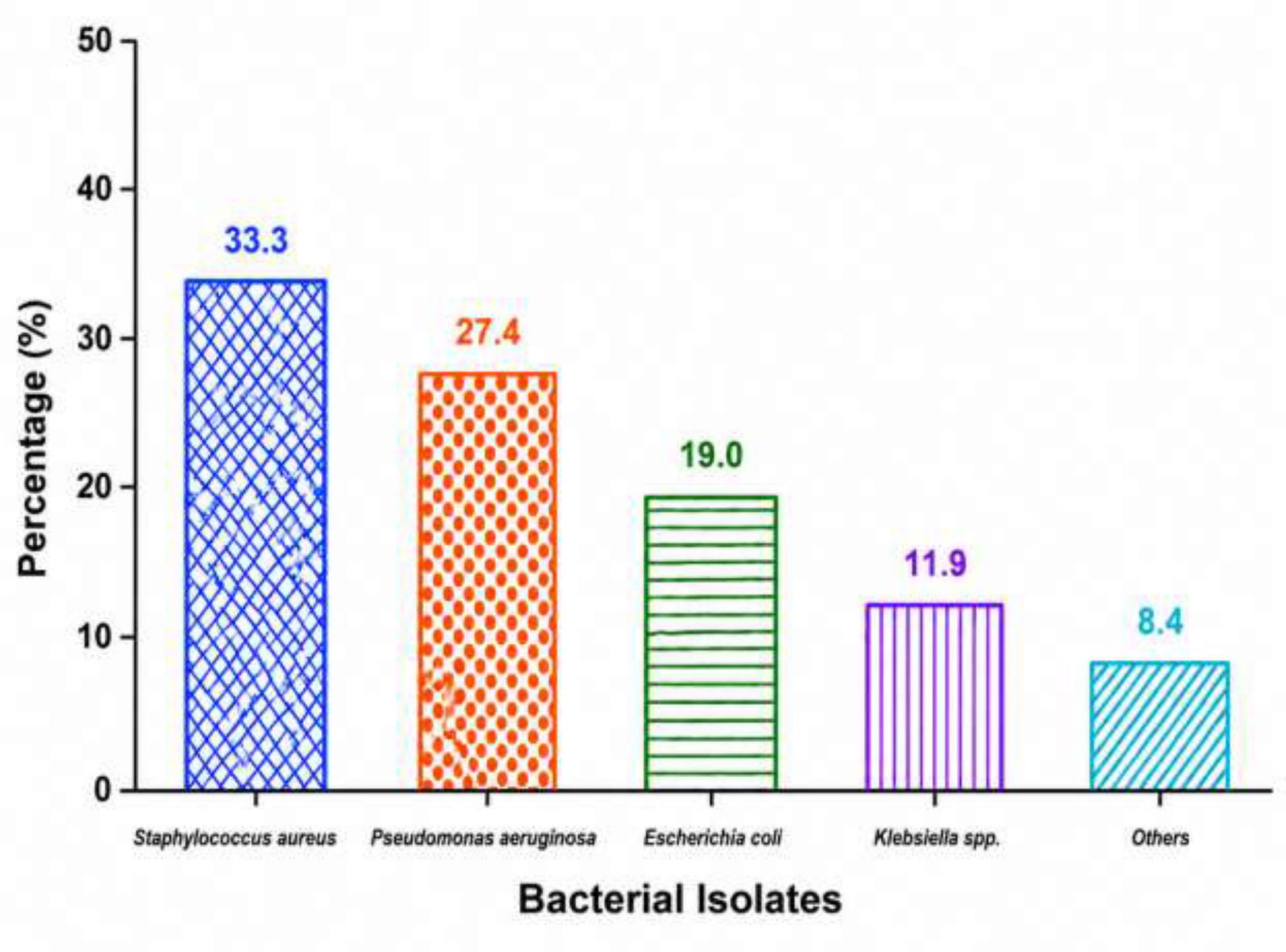
Distribution of bacterial species associated with wound infections. The graph shows the prevalence of major bacterial pathogens, with *Staphylococcus aureus* being the most predominant organism, followed by *Pseudomonas aeruginosa, Escherichia coli, Klebsiella* spp., and other species.

The most common pathogen identified was *Staphylococcus aureus* (33.3%), followed by *Pseudomonas aeruginosa* (27.4%), *Escherichia coli* (19.0%), and *Klebsiella* spp. (11.9%). Other bacterial species were grouped and accounted for 8.4% of the isolates (Figure 1).

### 3.2 Antimicrobial resistance and MIC profiles

**Figure 2:**
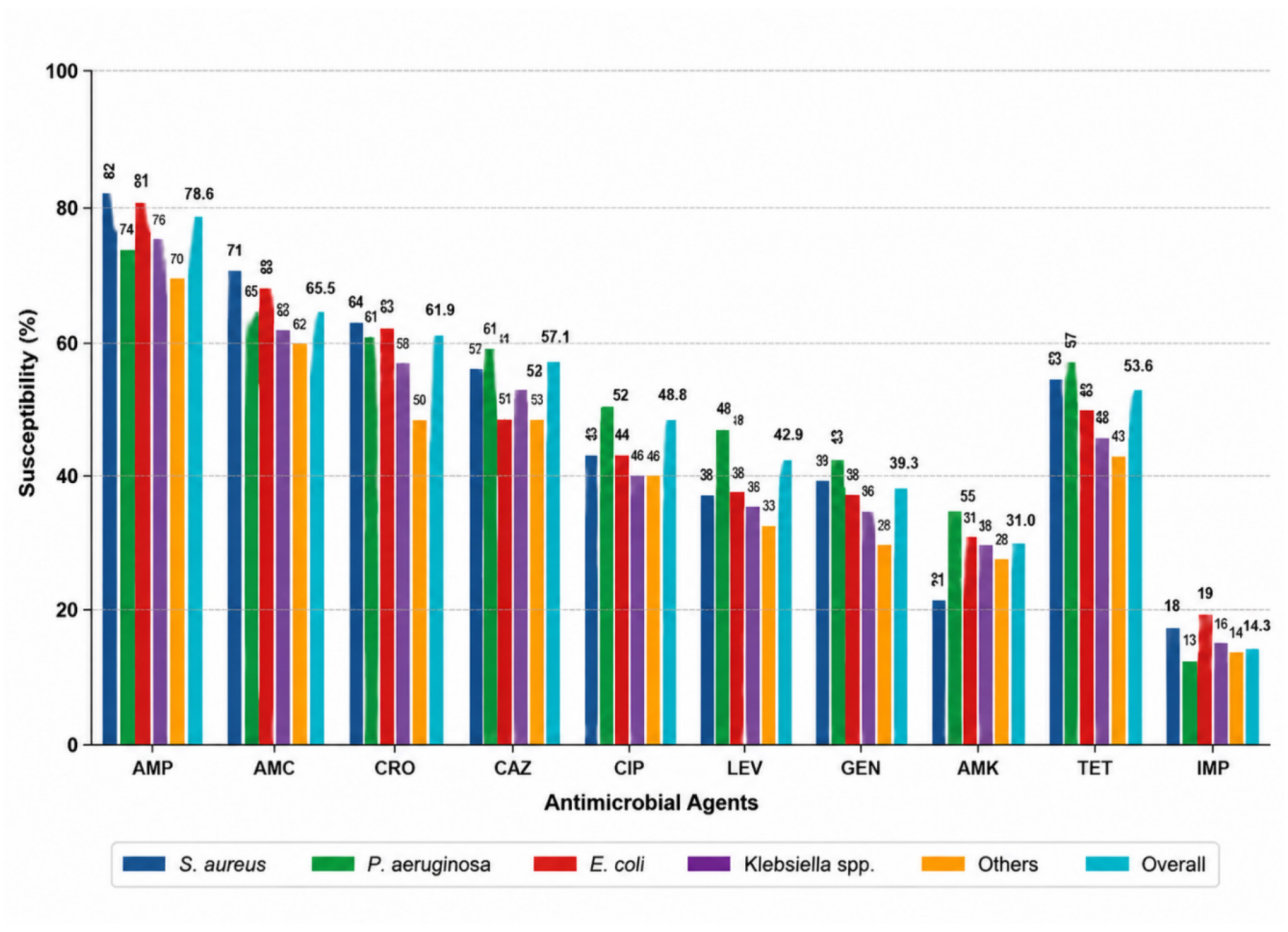
Antimicrobial susceptibility profile of bacterial isolates. The graph illustrates the percentage susceptibility of different bacterial isolates (*Staphylococcus aureus, Pseudomonas aeruginosa, Escherichia coli, Klebsiella* spp., and other organisms) against selected antimicrobial agents. The results demonstrate varied susceptibility patterns among bacterial species, reflecting differences in antimicrobial response and potential resistance profiles among wound-associated pathogens.

Antimicrobial susceptibility testing showed a high proportion of resistance in isolates, especially to β-lactam antibiotics. The highest overall resistance rates were observed for ampicillin (78.6%), amoxicillin-clavulanic acid (65.5%), ceftriaxone (61.9%), and ceftazidime (57.1%). Moderate resistance was observed for ciprofloxacin (48.8%), levofloxacin (42.9%), gentamicin (39.3%), and amikacin (31.0%). Imipenem exhibited the lowest resistance rate (14.3%), indicating retained effectiveness against most isolates (Table 2, Figure 2).

**Table 2:** Overall antibiotic resistance profile (%).

Organism	AMP	AMC	CRO	CAZ	CIP	LEV	GEN	AMK	TET	IMP
*S. aureus*	82	71	64	57	43	36	39	21	54	18
*P. aeruginosa*	74	65	61	61	52	48	43	35	57	13
*E. coli*	81	69	63	50	44	38	38	31	50	19
*Klebsiella spp*.	76	62	58	55	40	36	35	30	46	15
Others	70	60	50	50	40	33	30	28	43	14
Overall	78.6	65.5	61.9	57.1	48.8	42.9	39.3	31.0	53.6	14.3

**Figure 3:**
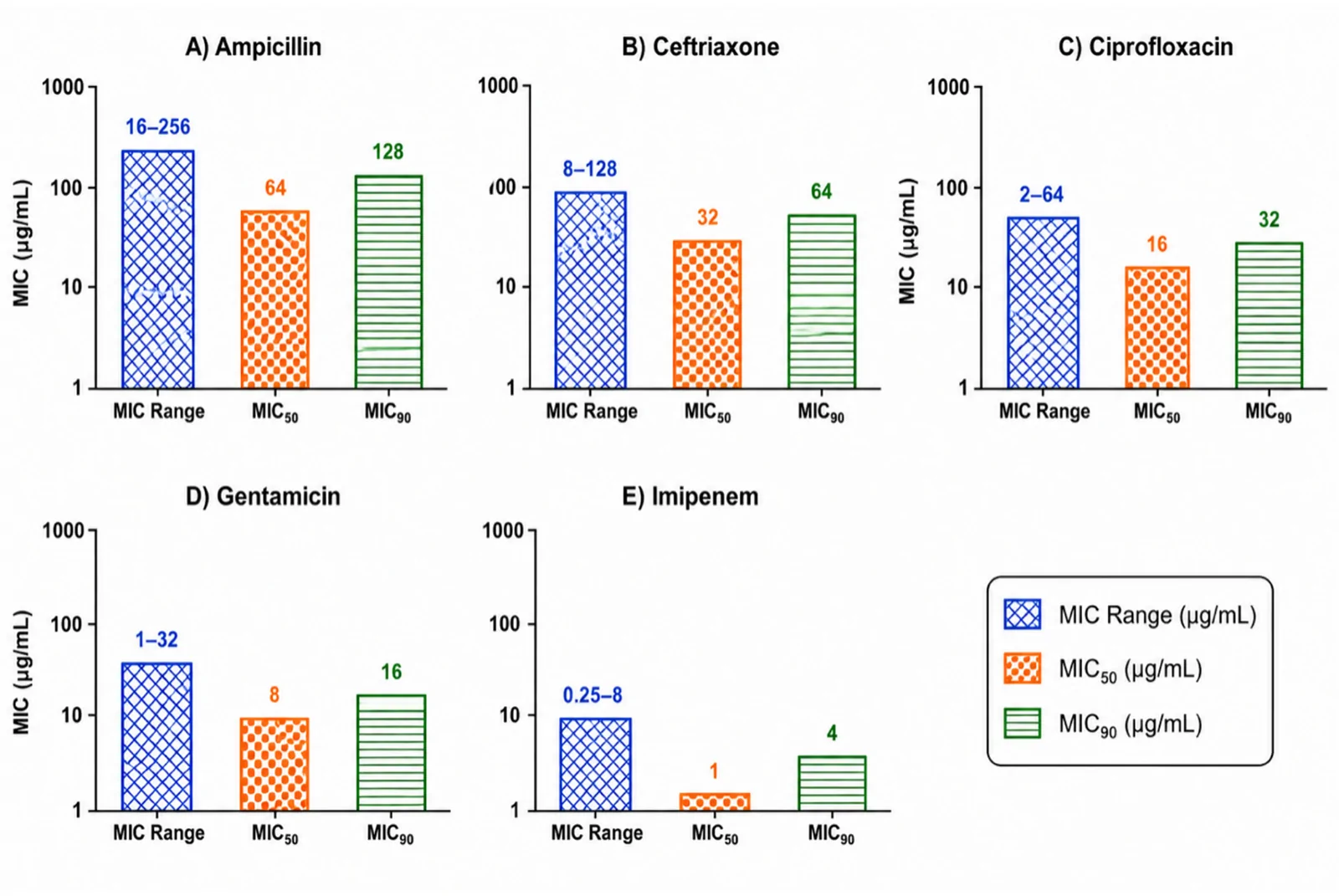
Minimum Inhibitory Concentration (MIC) profiles of five antibiotics against the tested bacterial isolates. Panels represent the results for **(A)** Ampicillin, **(B)** Ceftriaxone, **(C)** Ciprofloxacin, **(D)** Gentamicin, and **(E)** Imipenem. The vertical axis displays the MIC values on a logarithmic scale (μg/ml). Within each panel, the three bars denote the MIC range (blue bars with crosshatching), the concentration required to inhibit 50% of isolates (MIC50, orange bars with diamond pattern), and the concentration required to inhibit 90% of isolates (MIC90, green bars with horizontal lines). Specific numerical values for each metric are presented directly above the corresponding bars.

MIC analysis further confirmed reduced susceptibility among the isolates. Ampicillin demonstrated the highest MIC90 value (128 μg/ml), followed by ceftriaxone (64 μg/ml), ciprofloxacin (32 μg/ml), gentamicin (16 μg/ml), and imipenem (4 μg/ml). The findings indicate reduced antimicrobial susceptibility among the tested isolates. Higher MIC values were observed among isolates that also exhibited strong biofilm-forming ability; however, MIC measurements reflect planktonic bacterial susceptibility and do not directly represent biofilm-associated antimicrobial tolerance (Table 3, Figure 3).

**Table 3:** MIC distribution of selected antibiotics (μg/ml).

Antibiotic	MIC range	MIC50	MIC90
Ampicillin	16–256	64	128
Ceftriaxone	8–128	32	64
Ciprofloxacin	2–64	16	32
Gentamicin	1–32	8	16
Imipenem	0.25–8	1	4

### 3.3 Biofilm formation and association with multidrug resistance

**Figure 4:**
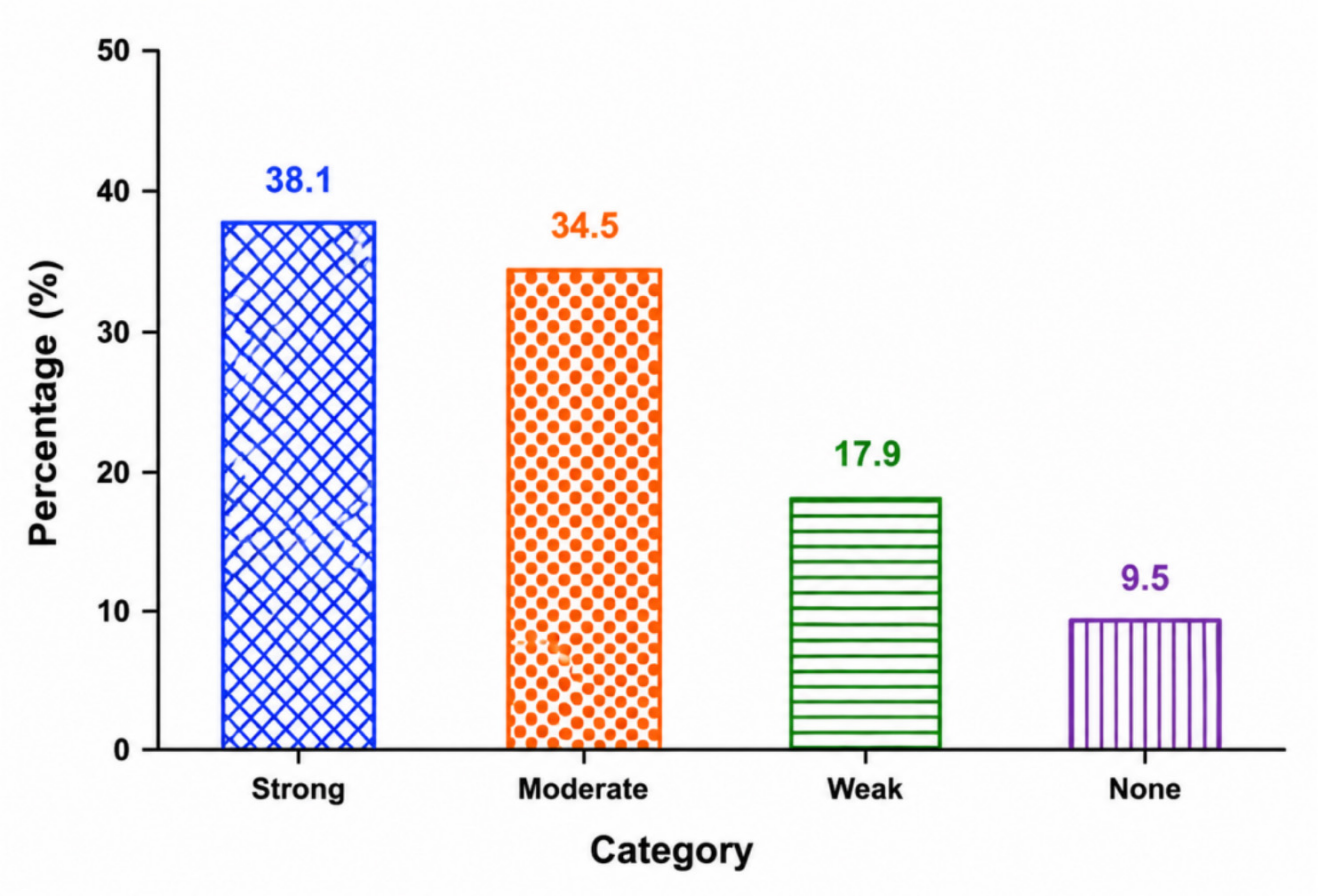
Biofilm formation categories among bacterial isolates, showing the distribution of strong, moderate, weak, and non-biofilm producers. Biofilm production was detected in most isolates. Strong producers comprised the largest group (*n* = 32), followed by moderate (*n* = 29), weak (*n* = 15), and non-producers (*n* = 8).

Biofilm production was detected among most bacterial isolates. Strong biofilm producers accounted for 38.1% of isolates (n = 32), followed by moderate (34.5%, *n* = 29), weak (17.9%, *n* = 15), and non-biofilm-producing isolates (9.5%, *n* = 8) (Figure 4).

**Figure 5:**
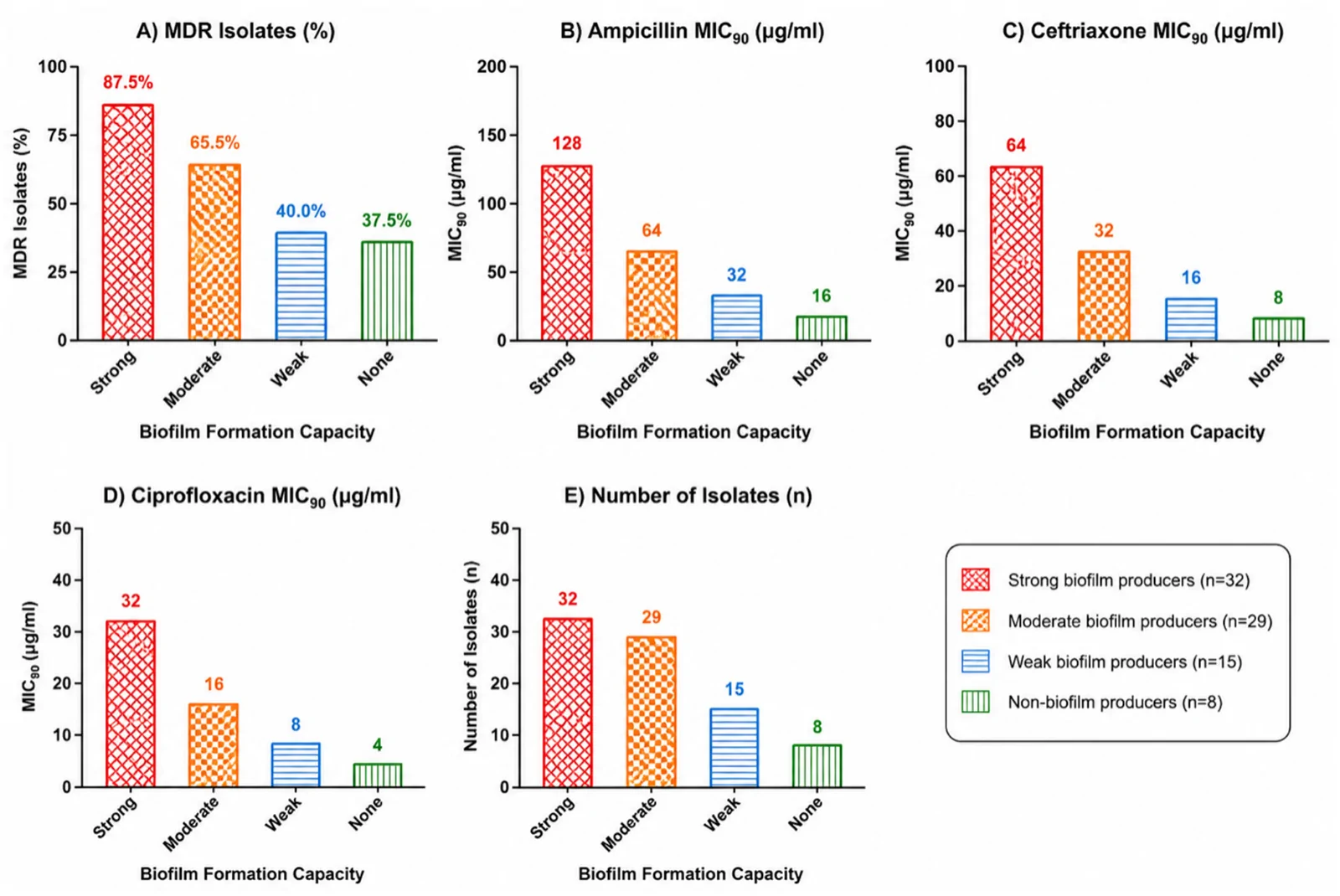
Relationship between biofilm formation capacity, multidrug resistance (MDR) prevalence, and antibiotic MIC90 values among bacterial isolates. Panels show **(A)** MDR prevalence (%), **(B)** Ampicillin MIC90, **(C)** Ceftriaxone MIC90, **(D)** Ciprofloxacin MIC90, and **(E)** the number of isolates across strong (*n* = 32), moderate (*n* = 29), weak (*n* = 15), and non-biofilm (*n* = 8) producers. Increasing biofilm-forming capacity was associated with higher MDR prevalence and elevated MIC90 values for all three antibiotics. MIC values represent planktonic bacterial susceptibility and should not be interpreted as direct measurements of biofilm-associated resistance.

Overall, 63.5% of isolates were classified as multidrug-resistant (MDR), defined as resistance to at least three antimicrobial classes. MDR prevalence increased with biofilm-forming ability, with the highest proportion observed among strong biofilm producers (87.5%). Strong biofilm-producing isolates also exhibited higher MIC90 values for ampicillin, ceftriaxone, and ciprofloxacin compared with moderate, weak, and non-biofilm producers (Table 4, Figure 5), indicating an association between enhanced biofilm formation and reduced antimicrobial susceptibility.

**Table 4:** Association between biofilm formation capacity, multidrug resistance prevalence, and MIC90 values among bacterial isolates.

Biofilm formation category	Number of isolates (*n*)	MDR isolates (%)	Ampicillin MIC90	Ceftriaxone MIC90	Ciprofloxacin MIC90
Strong biofilm producers	32	87.5	128	64	32
Moderate biofilm producers	29	65.5	64	32	16
Weak biofilm producers	15	40.0	32	16	8
Non-biofilm producers	8	37.5	16	8	4

**Figure 6:**
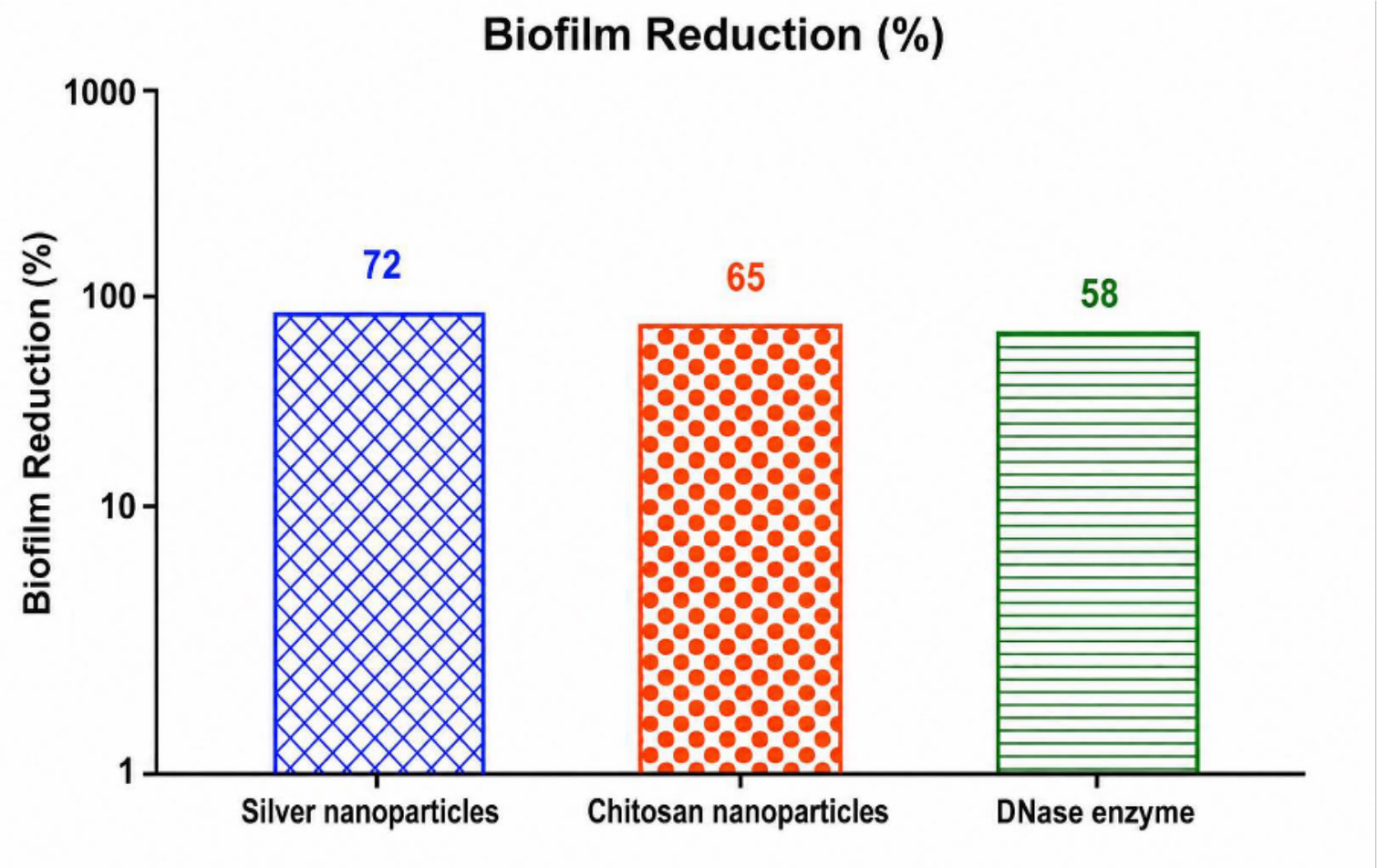
Biofilm reduction efficiency of different antimicrobial agents. The graph shows the percentage reduction of bacterial biofilms following treatment with silver nanoparticles, chitosan nanoparticles, and DNase enzyme. Silver nanoparticles exhibited the highest biofilm reduction (72%), followed by chitosan nanoparticles (65%) and DNase enzyme (58%), demonstrating their potential antibiofilm activity against wound-associated bacterial biofilms.

The anti-biofilm activity of the tested agents was evaluated against strong biofilm-producing isolates. Silver nanoparticles exhibited the highest biofilm reduction activity, reducing biofilm biomass by 72%. Chitosan nanoparticles showed a 65% reduction, while DNase enzyme treatment resulted in a 58% reduction compared with untreated controls (Figure 6).

These findings demonstrate that nanoparticle-based and enzyme-mediated approaches can effectively disrupt established biofilms and may represent potential strategies for managing biofilm-associated multidrug-resistant wound infections.

## 4 Discussion

Chronic wound infections represent a significant clinical challenge due to their complex microbial composition, high rates of antimicrobial resistance, and the ability of bacterial communities to form persistent biofilms. The present study investigated the bacterial profile, antimicrobial susceptibility patterns, biofilm-forming capacity, and anti-biofilm potential of different agents among bacterial isolates recovered from chronic wound infections. The high culture positivity rate (87.5%) observed in this study confirms the frequent involvement of bacterial pathogens in chronic wound complications. It supports previous reports describing chronic wounds as favorable environments for microbial colonization and infection development (13, 14).

In the present study, *Staphylococcus aureus* (33.3%) was the predominant bacterial isolate, followed by *Pseudomonas aeruginosa* (27.4%), *Escherichia coli* (19.0%), and *Klebsiella* spp. (11.9%). The predominance of *S. aureus* and *P. aeruginosa* is consistent with previous studies identifying these organisms as major pathogens associated with chronic wounds and soft tissue infections. Both species possess strong adaptive abilities, including biofilm formation, production of extracellular polymeric substances, and multiple antimicrobial resistance mechanisms, which contribute to their persistence in chronic wound environments (15). Similar bacterial distributions have been reported in diabetic foot ulcers and other non-healing wounds, where *S. aureus, P. aeruginosa*, Enterobacteriaceae, and other opportunistic pathogens frequently coexist (14, 16).

Biofilm formation is recognized as an important survival strategy among chronic wound pathogens. In this study, most isolates demonstrated biofilm-forming ability, with strong biofilm producers representing 38.1% of all isolates. Furthermore, a significant association was observed between biofilm-forming capacity and MDR prevalence, as strong biofilm-producing isolates showed a higher proportion of MDR phenotypes compared with weaker or non-biofilm producers. Previous studies have similarly reported that biofilm-producing bacteria are frequently associated with multidrug-resistant characteristics and prolonged infection persistence (15, 17). The present findings support the concept that biofilm formation and antimicrobial resistance commonly coexist among chronic wound isolates; however, the observed relationship should be interpreted as an association rather than a direct causal effect (18, 19).

In this study, strong biofilm-producing isolates also showed higher MIC90 values for selected antibiotics compared with isolates exhibiting lower biofilm-forming capacity. Since MIC measurements evaluate antimicrobial susceptibility of planktonic bacterial populations, these findings do not directly represent biofilm-associated antimicrobial tolerance. Instead, the observed relationship suggests that isolates with strong biofilm-forming ability may also possess additional resistance characteristics contributing to reduced antimicrobial susceptibility. Biofilm-associated mechanisms, including extracellular polymeric substance production, altered metabolic activity, quorum sensing regulation, and stress-response pathways, may contribute to enhanced survival during antimicrobial exposure (20, 21). Further studies using specific biofilm susceptibility assays, such as minimum biofilm inhibitory concentration (MBIC) or minimum biofilm eradication concentration (MBEC), are required to directly evaluate antimicrobial activity against established biofilms.

The elevated MIC values (2–4-fold increase) observed in strong biofilm producers in this study can be explained by multiple protective mechanisms inherent to biofilms. The extracellular polymeric substance (EPS) matrix creates a diffusion barrier limiting antibiotic penetration. Further, persisted cells in biofilms are metabolically inert and can be intrinsically resistant to antibiotics (22). Also, gene expression for virulence and resistance is regulated by quorum-sensing. The results are consistent with previous studies that have shown that the architecture of biofilms greatly contributes to antimicrobial resistance and survival of bacteria in the face of antimicrobial exposure (23, 24).

The anti-biofilm evaluation demonstrated that silver nanoparticles exhibited the highest biofilm reduction activity, achieving a 72% reduction in biofilm biomass, followed by chitosan nanoparticles (65%) and DNase treatment (58%). These findings support previous studies reporting the potential of nanomaterials and enzymatic approaches as promising strategies for disrupting biofilm structures (11, 25). Silver nanoparticles possess broad antimicrobial activity through multiple mechanisms, including membrane disruption, reactive oxygen species generation, protein dysfunction, and interference with bacterial genetic processes (26, 27). These multimodal effects may explain their strong biofilm-reducing activity observed in the present study.

Chitosan nanoparticles also demonstrated substantial anti-biofilm activity, which may be attributed to their positively charged surface interactions with bacterial membranes, leading to membrane destabilization and reduced bacterial adhesion. Similarly, DNase treatment showed biofilm-reducing activity by degrading extracellular DNA, an essential structural component of the biofilm matrix. Previous studies have demonstrated that enzymatic degradation of biofilm matrix components can weaken biofilm architecture and enhance bacterial susceptibility to antimicrobial interventions (28, 29).

From a clinical perspective, the findings highlight the importance of considering biofilm formation during chronic wound management. Biofilm-associated infections are often difficult to eradicate because bacteria within biofilm communities exhibit enhanced survival characteristics and reduced responsiveness to conventional antimicrobial treatments (30, 31). Therefore, combined therapeutic strategies integrating appropriate antimicrobial therapy with anti-biofilm approaches may provide improved outcomes for chronic wound infections.

The presence of MDR and biofilm-forming pathogens also has implications within the One Health framework, particularly considering the potential exchange of resistant microorganisms between humans, animals, and the environment. Appropriate antimicrobial use, infection control practices, and development of alternative anti-biofilm strategies are essential components of addressing the global antimicrobial resistance challenge (32, 33).

In conclusion, this study demonstrates a high prevalence of bacterial infection, substantial antimicrobial resistance, and widespread biofilm-forming ability among chronic wound isolates. The observed association between biofilm formation, MDR prevalence, and reduced antimicrobial susceptibility emphasizes the importance of integrated approaches targeting both bacterial resistance and biofilm development. Silver nanoparticles, chitosan nanoparticles, and DNase represent promising complementary strategies for biofilm reduction; however, further investigations using standardized biofilm susceptibility models and *in vivo* studies are required to determine their clinical applicability.

### 4.1 Future directions

Future studies should be directed toward finding more efficient and clinically relevant methods to deal with the problem of multidrug-resistant infections in chronic wounds associated with biofilms. Large-scale clinical trials are needed to establish the efficacy and safety of newly developed anti-biofilm agents, particularly formulations of silver nanoparticles or chitosan nanoparticles in real clinical settings. Furthermore, combination treatment using traditional antibiotics and biofilm-disrupting agents, enzymes (DNase), or quorum-sensing inhibitors could be used to enhance treatment. Improvements in molecular diagnostics and genomics could provide even greater improvements in the identification of biofilm-forming and resistant pathogens, thereby allowing targeted therapy. Zoonotic transmission of MDR biofilm-producing organisms and rational use of antimicrobials in both human and veterinary medicine, from a One Health perspective, should also be considered in future studies. Overall, an integrated approach that includes microbiology, nanotechnology, and clinical practice will be necessary to successfully treat and prevent chronic infections associated with biofilms.

## 5 Conclusion

In conclusion, the present study demonstrates that chronic wound infections are strongly associated with biofilm-forming bacteria, particularly *Staphylococcus aureus* and *Pseudomonas aeruginosa*, which exhibit a high prevalence of multidrug resistance. Increased antimicrobial resistance was significantly correlated with the production of biofilm, indicating the importance of biofilm in non-healing and persistent infections. Increased MICs for the strong biofilm producers further highlight the suboptimal effect of traditional antibiotic treatment. In addition, the study highlights silver nanoparticles, chitosan nanoparticles, and DNase as potential anti-biofilm agents that can effectively decrease the biofilm biomass. The results highlight the critical need for a combined therapeutic approach that includes antimicrobial treatment as well as strategies to disrupt biofilms. In conclusion, biofilm-mediated resistance has emerged as a multifaceted challenge in the field of chronic wound infections, highlighting the need for a comprehensive strategy that includes the use of new antimicrobial agents and therapies.

## Data Availability

The original contributions presented in the study are included in the article/supplementary material, further inquiries can be directed to the corresponding author.
